# Supplementary material for: A set of multi-entry identification keys to African frugivorous flies (Diptera, Tephritidae)
Source: Zookeys. 2014 Jul 24;(428):97–108. doi: 10.3897/zookeys.428.7366 (PMC4143993; doi:10.3897/zookeys.428.7366)
Supplement: Supplementary material 4 — Key to Capparimyia [file zookeys-428-097-s004.zip › SF4_ZooKeys_key to Capparimyia/lucid_player/help/help/why_discarded.htm]

Why an entity was discarded - The Why Discarded function


# Why an entity was discarded - The Why Discarded function

After choosing several states, entities which do not match the current
selection are moved to the *Entities
Discarded* window. To determine why an entity was discarded, select
it from the Entities Discarded window then select *Why
Discarded* from the *Entities*
menu or click on the *Why Discarded*
button on the toolbar.

The *Why Discarded* dialog which
opens displays the list of chosen features that do not match the selected
entity. For each feature, the list of states that you chose from the feature
are displayed, along with the list of states present for the entity in
the key.
  
 
  
If
an entity is discarded that you believe should still be present, the *Why Discarded* function allows you to
quickly compare your selections with the features present for the entity
in the key.
